# The effects of acupuncture therapy in migraine: An activation likelihood estimation meta-analysis

**DOI:** 10.3389/fnins.2022.1097450

**Published:** 2023-01-27

**Authors:** Jing Zhao, Liu-xue Guo, Hong-ru Li, Xin-yun Gou, Xiao-bo Liu, Yue Zhang, Dong-ling Zhong, Yu-xi Li, Zhong Zheng, Juan Li, Yue Feng, Rong-jiang Jin

**Affiliations:** ^1^School of Health Preservation and Rehabilitation, Chengdu University of Traditional Chinese Medicine, Chengdu, Sichuan, China; ^2^Department of Critical Care Medicine, Hospital of Chengdu University of Traditional Chinese Medicine, Chengdu, Sichuan, China; ^3^Centre of Preventive Treatment of Disease, Hospital of Chengdu University of Traditional Chinese Medicine, Chengdu, Sichuan, China; ^4^Mental Health Center, West China School of Medicine, West China Hospital, Sichuan University, Chengdu, Sichuan, China; ^5^School of Acupuncture and Tuina, Chengdu University of Traditional Chinese Medicine, Chengdu, Sichuan, China

**Keywords:** acupuncture, migraine, functional magnetic resonance imaging, meta-analysis, activation likelihood estimation

## Abstract

**Background:**

Previous functional magnetic resonance imaging studies indicated that acupuncture could activate the brain regions in patients with migraine. However, these studies showed inconsistent results. This activation likelihood estimation (ALE) meta-analysis aimed to investigate the consistent activated change of brain regions between pre- and post-acupuncture treatment in migraineurs.

**Methods:**

We conducted a literature search in PubMed, Embase, Web of Science, the Cochrane Library, the China National Knowledge Infrastructure, the Chinese Science and Technology Periodical Database, the Wanfang Database, and the Chinese Biomedical Literature Database from their inception to 18 August, 2022, to obtain articles assessing the functional magnetic resonance imaging changes of acupuncture for migraine. Two investigators independently performed literature selection, data extraction, and quality assessment. The methodological quality was assessed with a modified version of the checklist. The reporting quality of interventions among included studies was evaluated by the Revised Standards for Reporting Interventions in Clinical Trials of Acupuncture (STRICTA). Our meta-analysis was conducted according to the GingerALE software. The Jackknife sensitivity analysis was used to assess the robustness of the results.

**Results:**

14 articles were finally included according to the eligible criteria. Regarding the immediate effect of acupuncture on migraine, the ALE meta-analysis demonstrated that the deactivation regions were mainly located in the superior frontal gyrus, and middle frontal gyrus (uncorrected *P* < 0.001). The ALE meta-analysis of the cumulative effect showed that the activation regions were the thalamus, superior frontal gyrus, posterior lobe of the cerebellum, insula, middle frontal gyrus, precentral gyrus, anterior cingulate, and the deactivation brain regions were located in the transverse temporal gyrus, postcentral gyrus, superior temporal gyrus, anterior cingulate, parahippocampal gyrus, inferior parietal lobule, and inferior occipital gyrus (uncorrected *P* < 0.001).

**Conclusion:**

Acupuncture could activate multiple brain areas related with the regulation of pain conduction, processing, emotion, cognition, and other brain regions in patients with migraine. In the future, the combination of multiple imaging technologies could be a new approach to deeply investigate the central mechanism of acupuncture for migraine.

## 1. Introduction

Migraine is a chronic paroxysmal neurological disorder, along with multiphase attacks of headache and a myriad of neurological symptoms (Dodick, 2018). According to epidemiological statistics (Safiri et al., 2022), the global age-standardized point prevalence and annual incidence rate of migraine were 14,107.3 and 1,142.5 per 100,000 in 2019, and migraine prevalence peaks in 40 to 44 age group. Migraine sufferers have a variety of problems that can lead to decreased productivity at school, at home, and in society. Globally, migraine is the leading cause of years lived with disability (YLDs) accounting for 45.1 million YLDs annually (Gbd 2016 DALYs and Hale Collaborators, 2017). The economic costs of migraine are substantial. The high prevalence and societal burden of migraine have contributed to the acknowledgment of migraine as a serious public health concern. At present, pharmaceutical treatment for migraine is the most basic and common treatment. However, the efficacy of most existing drugs for migraine is limited, and long-term use may produce significant side effects, such as addiction, and overdose deaths (Hagemeier, 2018). For the management of migraine, safe, effective, and acceptable non-pharmacological treatments are crucial to solve this problem.

Mounting evidence supports the application of acupuncture to prevent and treat migraines, due to its long-term effectiveness, good tolerance, and fewer side effects. A Cochrane review from Germany concluded that acupuncture was effective and safe for episodic migraine prophylaxis when compared with prophylactic drug treatment (Linde et al., 2016). Apart from reducing pain, acupuncture may be beneficial for migraineurs with co-morbid problems such as anxiety, insomnia, and muscle tension (Liao et al., 2020; Natbony and Zhang, 2020). Acupuncture imaging studies have confirmed that the anti-migraine mechanism of acupuncture is closely related to the regulation of cerebral activities, which can modulate the functional and structural brain in patients with migraine (Ma et al., 2021). Li et al. (2017) found that acupuncture could normalize the decreased amplitude of low-frequency fluctuation (ALFF) of the rostral ventromedial medulla/trigeminocervical complex in migraineurs. Liu S. et al. (2021) observed that the region homogeneity (ReHo) values in the cerebellum and angular gyrus increased significantly after 12 sessions of acupuncture treatment in patients with migraine. Zhang et al. (2016) discovered that acupuncture could increase the functional connectivity (FC) of brain regions in patients with migraine. Nevertheless, the underlying central mechanism of acupuncture for migraine is still not completely studied.

The resting-state fMRI (functional magnetic resonance imaging) is used to measure the spontaneous activity of neurons by enabling the recording of the blood oxygenation level dependent (BOLD) signals. Three indicators are commonly used to determine spontaneous brain activity: ALFF, fractional ALFF (fALFF), and ReHo (Zang et al., 2004; Liu S. et al., 2022). ALFF and fALFF reflect the regional intensity of spontaneous fluctuations in the BOLD signal (Zang et al., 2007; Zou et al., 2008). ReHo indicates the synchronization and consistency of the BOLD signal between a single voxel and neighboring voxels (Zang et al., 2004). Therefore, the combination of ALFF, fALFF, and ReHo can fully present spontaneous activity of the local brain. Activation likelihood estimation (ALE) is an effective method for meta-analysis of brain neuroimaging, which is a brain region localization analysis method based on voxel coordinates, and the brain region localization can be achieved by carrying out 3D Gaussian smoothing and permutation tests of the relevant coordinates in the included studies. Previous reviews narratively summarized the functional brain changes of acupuncture in patients with migraine (Liu L. et al., 2021; Ma et al., 2021), while quantitative meta-analyses were not performed. Therefore, the purpose of this study was to explore the acupuncture-related brain regions in migraineurs with ALE algorithm.

## 2. Methods

The protocol of this ALE-meta analysis has already been registered on the International Platform of Registered Systematic Review and Meta-analysis Protocols (INPLASY)^1^ (registration number: INPLASY2022110026). The present study was reported in accordance with the Preferred Reporting Items for Systematic Reviews and Meta-Analyses (PRISMA) statement (Page et al., 2021).

### 2.1. Literature search

We performed a comprehensive search in the following databases from their inception to August 18, 2022: PubMed, EMBASE, Web of Science, the Cochrane Library, the China National Knowledge Infrastructure (CNKI), the China Science and Technology Journal Database (VIP), Wanfang Database, and the China Biology Medicine (CBM). Both Medical Subject Headings (MeSH) and free-text words related to acupuncture, migraine, and fMRI were used to retrieve relevant studies. We additionally searched the references of the included studies. We consulted the specialists for possible eligible studies. The full search strategies for all databases are shown in Supplementary Table 1.

### 2.2. Inclusion criteria

We included studies that met the following criteria:

1) The patients were diagnosed with migraine by any internationally recognized or accepted clinical guideline or consensus like The International Classification of Headache Disorders, 3rd edition (beta version) (Headache Classification Committee of the International Headache Society (IHS), 2013);
2) The intervention involved electro-acupuncture or manual acupuncture; no limitations on manipulation methods of acupuncture, acupoint selection, and duration of acupuncture;
3) The studies reported neuroimaging results (ReHo, ALFF, or fALFF) of pre- and post-acupuncture treatment *via* fMRI using the standard anatomical template;
4) Both randomized controlled trials and clinical controlled trials were included.

### 2.3. Exclusion criteria

We excluded studies that fulfilled the following criteria:

1) No detailed description of the diagnostic criteria;
2) Full texts were unavailable through extensive search;
3) Coordinates could not be obtained through various approaches;
4) The results were based on the region of interest.

### 2.4. Study selection

The retrieved records were imported into Endnote (X9). After removing duplicates, two researchers (X-BL and X-YG) independently eliminated irrelevant records by reading the titles and abstracts, then screened the rest records in full text to identify eligible studies. After selection, two reviewers cross-checked, and disagreements were settled through team discussion or consultation with the third reviewer (JL).

### 2.5. Data extraction

Two independent reviewers (X-BL and X-YG) extracted the following information: (1) publication information: title, first author, year of publication; (2) demographic characteristics: types of migraine, diagnostic criteria, sample size, characteristics of the study population (age, gender); (3) intervention details: manipulation methods of acupuncture, frequency, duration, and sessions; (4) neuroimaging data: MRI acquisition, processing parameters, analysis parameters, activation coordinates (POST > PRE), and deactivation coordinates (PRE > POST), along with their associated standard anatomical template. If a study observed the neuroimaging results of different acupoints or needle stimulations, we extracted data separately. After extraction, two reviewers cross-checked to ensure accuracy. Any disagreement was resolved through discussion or arbitration by a third reviewer (JL).

### 2.6. Assessment of methodological quality

A modified version of checklist (Iwabuchi et al., 2015; Pan et al., 2017) was used to assess the methodological quality of individual functional neuroimaging studies. The checklist contains two domains (Category 1: Sample characteristics, Category 2: Methodology and reporting) with 13 items. The overall score is 20 points. The higher the score, the better the methodological quality. Before the formal evaluation, two reviewers intensively discussed the checklist to achieve consensus. Then two independent reviewers (X-BL and X-YG) assessed and cross-checked the results. Discrepancies were resolved by team discussion.

### 2.7. Evaluation of reporting quality of interventions in controlled trials of acupuncture

The Revised Standards for Reporting Interventions in Clinical Trials of Acupuncture (STRICTA) was used to appraise the reporting quality of interventions in controlled trials of acupuncture (Macpherson et al., 2010). The STRICTA consists of six items (17 sub-items), including acupuncture rationale, details of needling, treatment regimen, co-interventions, practitioner background, and control or comparator interventions. Then the two independent reviewers (X-BL and X-YG) assessed and cross-checked the results. Discrepancies were resolved by a third reviewer (JL).

### 2.8. Statistical analysis

**FIGURE 1:**
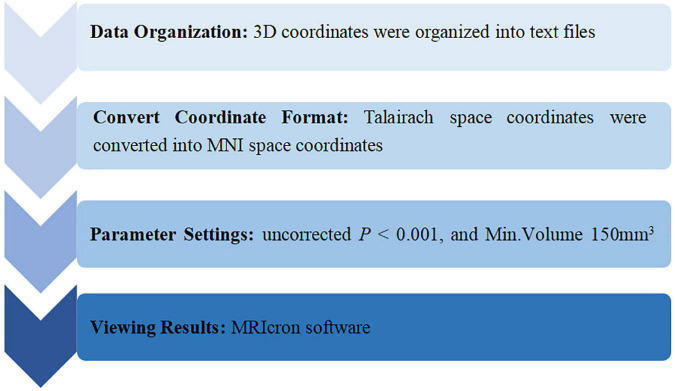
The process of ALE analysis.

The ALE meta-analysis was performed with the Ginger ALE software (version 2.3.6)^2^. The data analysis process was as follows: (1) Data organization: 3D (X, Y, Z) coordinates in the standard space included were organized into text files. (2) Convert coordinate format: Talairach space coordinates were converted into Montreal Neurological Institute (MNI) space coordinates using Ginger ALE software. (3) Parameter settings: uncorrected *P* < 0.001, and Min.Volume 150 mm^3^ (Yuan et al., 2022). (4) Viewing results: the results were presented in an Excel spreadsheet, and the ALE maps were overlaid onto the MNI template and viewed with MRIcron^3^. The process of data analysis is presented in Figure 1.

### 2.9. Sensitivity analysis

The reproducibility of the ALE meta-analysis results was assessed using the Jackknife sensitivity analysis method. That is, all studies were excluded one by one, and the remaining studies were re-analyzed.

## 3. Results

### 3.1. Search selection

**FIGURE 2:**
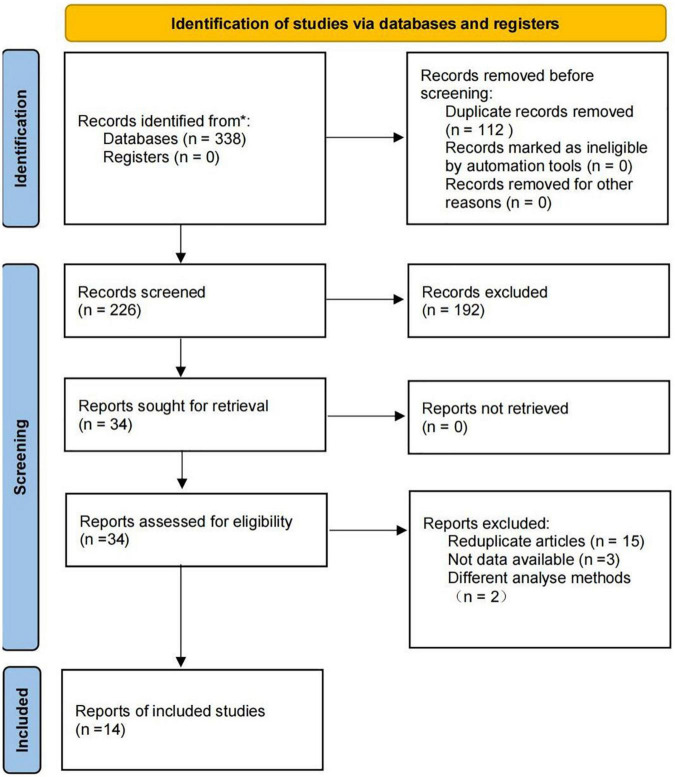
Flow diagram of literature search.

The flow diagram of study selection is shown in Figure 2. A total of 338 records were retrieved in the literature search. Following the removal of 112 duplicates, 212 irrelevant records were eliminated based on their titles and abstracts, and 20 records were excluded based on full-text screening. Finally, we identified 14 eligible studies. The list of excluded records with reasons is provided in Supplementary Table 2.

### 3.2. Study overview

We identified 14 eligible studies (Peng, 2013; Zhao et al., 2014; Xie, 2016; Zhang, 2016; Cai, 2017; Li et al., 2017; Ning et al., 2017; Du, 2019; Wang, 2019; Zhang, 2019; Fan, 2020; Jia, 2021; Liu S. et al., 2021; Zhang Y. et al., 2021) for this ALE meta-analysis, of which 9 were in Chinese and 5 in English. There were 9 randomized controlled trials and 5 non-randomized controlled trials. With regards to the subtypes of migraine patients, 4 studies included patients with menstrual migraine, 4 studies enrolled migraine patients without aura, and 6 studies did not describe the subtypes of migraine. As for the analytical method, 3 studies used both the ALFF and ReHo, 8 studies adopted the ReHo, and 3 studies applied the ALFF. The details of all the included studies are presented in Table 1.

**TABLE 1:** Characteristics of included studies.

References	Disease	Study type	Sample size		Age (years ± SD)		Gender (M/F)		Experiments	Analytical method	Standard anatomical template
			**AG**	**CG** **(Patient/HC)**	**Patient**	**HC**	**Patient**	**HC**			
Peng, 2013	Migraine	Non-RCT	32	18 (0/18)	37.91 ± 5.354	37.5 ± 5.272	16/16	9/9	1	ReHo	Talariach
Zhao et al., 2014	Migraine	RCT	20	20 (20/0)	AG:32.90 ± 10.99 CG: 37.25 ± 9.68	–	14/26	–	1	ReHo	Talariach
Xie, 2016	Migraine without aura	RCT	19	19 (19/0)	AG:29.58 ± 6.26 CG: 30.52 ± 6.85	–	8/30	–	2	ReHo	MNI
Zhang, 2016	Migraine	Non-RCT	20	20 (0/20)	21.70 ± 2.29	21.40 ± 0.82	6/14	6/14	1	ReHo	Talariach
Cai, 2017	Menstrual migraine without aura	RCT	15	13 (13/0)	AG:31.80 ± 6.70 CG: 34.69 ± 7.51	–	0/28	–	1	ReHo	Talariach
Li et al., 2017	Migraine without aura	RCT	35	69 (27/42)	21.29 ± 0.44	21.21 ± 0.28	48/14	34/8	1	ALFF	MNI
Ning et al., 2017	Migraine without aura	Non-RCT	16	(0/16)	28.3 ± 6.0	27.1 ± 4.8	3/13	3/13	1	ALFF	MNI
Du, 2019	Menstrual migraine without aura	Non-RCT	PMM:10 MRM:29	–	PMM: — MRM: 32.59 ± 7.15	–	PMM:0/10 MRM:0/29	–	2	ALFF ReHo	Talairach
Wang, 2019	Menstrual migraine	RCT	AG1:8 AG2:7	12 (0/12)	–	28.22 ± 8.524	0/15	0/12	2	ALFF ReHo	Talairach
Zhang, 2019	Migraine without aura	RCT	20	20 (20/0)	AG:38.95 ± 12.61 CG:36.30 ± 9.56:	–	4/36	–	1	ReHo	MNI
Fan, 2020	Migraine	RCT	AG1:6 AG2:6	–	AG1: 37.00 ± 8.81 AG2: 43.83 ± 12.09	–	2/10	–	2	ALFF	MNI
Jia, 2021	Migraine	RCT	AG1:16 AG2:19	–	AG1: 38.88 ± 14.77 AG2: 33.45 ± 10.81	–	8/27	–	2	ReHo	MNI
Liu S. et al., 2021	Migraine Without Aura	Non-RCT	37	15 (0/15)	37.97 ± 9.82	34.88 ± 6.66	6/31	2/13	2	ReHo	MNI
Zhang Y. et al., 2021	Menstrual migraine	RCT	24	20 (20/0)	AG:33.04 ± 6.43 CG: 35.30 ± 9.43	–	0/44	–	1	ALFF ReHo	Talairach

MNI, Montreal Neurological Institute; RCT, randomized controlled trial; Non-RCT, non-randomized controlled trial; ALFF, amplitude of low-frequency fluctuation; fALFF, fractional amplitude of low-frequency fluctuation; ReHo, regional homogeneity; AG, acupuncture group; CG, control group; MA, manual acupuncture; EA, electro-acupuncture; SA, sham acupuncture; HC, health control; PMM, pure menstrual igraine; MRM, menstrually related migraine; F/M: female/male.

The needle stimulations were manual acupuncture (13 studies) and electro-acupuncture (1 study). There were 11 studies focused on the long-term efficacy of acupuncture for migraine and 5 studies on the immediate efficacy of acupuncture for migraine. Four studies included acupuncture with different acupoint protocols. The details of acupuncture treatment are shown in Table 2.

**TABLE 2:** Intervention details of included studies.

References	Study type	Interventions		Needle sessions	Needle duration	Needle frequency	Acupoints
		**AG**	**CG**				
Peng, 2013	Non-RCT	MA	–	Instant	–	–	Qiuxu (GB40)
Zhao et al., 2014	RCT	MA	SA	32	8 weeks	4 times per week	Fengchi (GB20), Yanglingquan (GB34), Qiuxu (GB40), Waiguan (SJ5)
Xie, 2016	RCT	MA	SA	20	4 weeks	5 times per week	Headache point
Zhang, 2016	Non-RCT	MA	HC	20	4 weeks	once per day	Yanglingquan (GB34), Qiuxu (GB40), Waiguan (SJ5)
Cai, 2017	RCT	MA	SA	27	3 months	Before menses (1 week):3 times per week; Onset of menses and after menses (2 weeks): 2 times per week	Fengchi (GB20), Shuaigu (GB8), Sanyinjiao (SP6), Neiguan (PC6), Taichong (LR3)
Li et al., 2017	RCT	MA	SA, WA, HC	20	4 weeks	5 times per week	AG1: Yanglingquan (GB34), Qiuxu (GB40), Waiguan (SJ5). AG2: Xiyangguan (GB33), Diwuhui (GB42), Sanyangluo (SJ8) AG3: Zusanli (ST36), Chongyang (ST42), Pianli (L16)
Ning et al., 2017	Non-RCT	MA	HC	Instant	–	–	Zulinqi (GB41)
Du, 2019	Non-RCT	MA	–	27 ± 6	3 menstrual cycles	Before menses (1 week):3 times per week; Onset of menses and after menses (2 weeks): 2 times per week	Fengchi (GB20), Shuaigu (GB8),Sanyinjiao (SP6), Neiguan (PC6),Taichong (LR3)
Wang, 2019	RCT	MA	HC	27 ± 6	3 menstrual cycles	Before menses (1 week):3 times per week; Onset of menses and after menses (2 weeks): 2 times per week	AG1: Fengchi (GB20), Shuaigu (GB8), Taichong (LR3) AG2: Fengchi (GB20), Shuaigu (GB8), Neiguan (PC6)
Zhang, 2019	RCT	MA	SA	12	4 weeks,	3 times per week	Benshen (GB13), Shuaigu (GB8), Fengchi (GB20), Baihui (DU20), Shenting (DU24)
Fan, 2020	RCT	MA	–	Instant	–	–	AG1: Naokong (GB19), Fengchi (GB20), Naohu (DU17), Fengfu (DU16) AG2: Shenting (DU24), Yintang (EX-HN3), Meichong (BL3), Cuanzhu (BL2), Toulinqi (GB15),Yuyao (EX-HN4),Touwei (ST8), Sizhukong (SJ23), Hanyan (GB4), Xuanli (GB6)
Jia, 2021	RCT	MA	–	12	4 weeks	3 times per week	AG1:Zuqiaoyin (GB44), Lidui (ST45), Zhiyin (BL67), ashi point AG2: ashi point
Liu S. et al., 2021	Non-RCT	EA	HC	12	6 weeks	2 times per week	Baihui (DU20), Taiyang (EX-HN5), Fengchi (GB20), Shuaigu (GB8), Xuanlu (GB5), Toulinqi (GB15), Hegu (LI4), Taichong (LR3)
Zhang Y. et al., 2021	RCT	MA	SA	27 or 27 ± 6	3 months	Before menses (1 week):3 times per week; Onset of menses and after menses (2 weeks): 2 times per week	Fengchi (GB20), Shuaigu (GB8), Neiguan (PC6), Sanyinjiao (SP6), Taichong (LR3)

RCT, randomized controlled trial; Non-RCT, non-randomized controlled trial; MA, manual acupuncture; EA, electro-acupuncture; SA, sham acupuncture; HC, health control; AG, acupuncture group; CG, control group.

Neuroimaging data was acquired at either 1.5 T (1 study) or 3 T (12 studies), while one study did not specify the magnetic field strength. Most of the studies used the Siemens MRI scanner (8 studies), others included General Electric (4 studies), United Imaging Medical Systems (1 study), and 1 study did not report the scanner. Most of the structural images were obtained using fast spoiled gradient sequence (9 studies), magnetization-prepared rapid acquisition with gradient echo sequence (MPRAGE) (2 studies), and 3 studies did not specify the T1. T2-functional images were mainly from the echo-planar imaging (EPI) sequence (12 studies). Statistical analysis was conducted in either statistical parametric mapping (SPM) (13 studies) or data processing assistant for resting-state fMRI (DPARSF) (1 study). The details of MRI acquisition and analysis are demonstrated in Table 3.

**TABLE 3:** The details of MRI acquisition and analysis.

References	MRI acquisition				T1				T2				Analysis	
	**Teslas**	**MRI-system**	**MRI-model**	**Head-coil**	**Sequence**	**TR (ms)**	**TE (ms)**	**Voxel size (mm)**	**Sequence**	**TR (ms)**	**TE (ms)**	**Voxel size (mm)**	**Software**	**Method**
Peng, 2013	3T	Siemens	Trio	–	FSPGR	1900	2.26	1 × 1 × 1	–	2000	30	–	SPM5	ReHo
Zhao et al., 2014	3T	Siemens	Allegra	8	–				EPI	2000	30	–	SPM5	ReHo
Xie, 2016	1.5T	Siemens	Sonata	standard	MPRAGE	24	6	–	EPI	2000	30	–	SPM8	ReHo
Zhang, 2016	3T	Siemens	–	–	FSPGR	1900	2.26	–	EPI	2000	30	–	SPM8	ReHo
Cai, 2017	3T	GE	MR750	12	FSPGR	2530	3.4	–	GRE-EPI	2000	30	3.75 × 3.75 × 4	SPM8	ReHo
Li et al., 2017	3T	Siemens	Trio Tim	8	FSPGR	1900	2.26	1 × 1 × 1	EPI	2000	30	–	SPM12	ALFF
Ning et al., 2017	3T	Siemens	Sonata	–	–	1900	2.52	–	–	2000	30	–	SPM8	ALFF
Du, 2019	3T	GE	MR750	–	FSPGR	–	–	–	GRE-EPI	2000	25	3.75 × 3.75 × 4	SPM8	ALFF/ ReHo
Wang, 2019	3T	GE	MR750	32	FSPGR	2530	3.4	1 × 1 × 1	GRE-EPI	2000	25	3.75 × 3.75 × 4	SPM12	ALFF/ ReHo
Zhang, 2019	3T	Siemens	Skyra	20	–	–	–	–	EPI	3000	30	2.3 × 2.3 × 3	SPM8	ReHo
Fan, 2020	–	–	–	12	FSPGR	2530	3.4	–	GRE-EPI	2000	30	2.3 × 2.3 × 5	SPM12	ALFF
Jia, 2021	3T	Siemens	Skyra	–	MPRAGE	2530	2.98	1 × 1 × 1	EPI	2000	30	3.5 × 3.5 × 3.5	DPARSF	ReHo
Liu S. et al., 2021	3T	United Imaging Medical Systems	uMR780	12	FSPGR	7.2	3.1	1 × 1 × 1	EPI	2000	30	–	SPM12	ReHo
Zhang Y. et al., 2021	3T	GE	MR750	8	FSPGR	–	–	–	EPI	2000	25	3.44 × 3.44 × 4	SPM12	ALFF/ ReHo

MRI, magnetic resonance imaging; GE, gradient echo pulse; FSPGR, fast spoiled gradient sequence; MPRAGE, magnetization-prepared rapid acquisition with gradient echo sequence; SPGR, spoiled gradient recalled sequence; SPM, statistical parametric mapping; fALFF, fractional amplitude of low-frequency fluctuation; ALFF, amplitude of low-frequency fluctuation; ReHo, regional homogeneity; TR, repetition time for the whole pulse sequence in MRI; TE, echo time i.e., Time between middle of exciting RadioFrequency pulse and middle of spin echo production; data processing assistant for resting-state fMRI (DPARSF).

### 3.3. Assessment of methodological quality

The methodological quality of included studies ranges from 16 to 19 points. In Category 1 (Sample characteristics), the patients from all included studies were evaluated with specific standardized diagnostic criteria (item 1), and all the important demographic data (item 2) were reported. While only 6 studies (Peng, 2013; Zhang, 2016; Li et al., 2017; Ning et al., 2017; Wang, 2019; Liu S. et al., 2021) recruited healthy comparison subjects and provided demographic data (item 3), only 1 study (Jia, 2021) reported important clinical variables (item 4), and the sample size per group of 11 studies (Peng, 2013; Zhao et al., 2014; Xie, 2016; Zhang, 2016; Cai, 2017; Li et al., 2017; Ning et al., 2017; Zhang, 2019; Jia, 2021; Liu S. et al., 2021; Zhang Y. et al., 2021) were > 10 (item 5). In Category 2 (Methodology and reporting), all the items were adequately reported. The detailed information of the methodological quality of included studies are shown in Supplementary Table 3. The modified version of checklist is provided in Supplementary Table 4.

### 3.4. STRICTA checklist for the included studies

According to the STRICTA checklist, the items with 70% of reporting rates were item 1a (style of acupuncture, 100%), item 1b (reasoning for treatment provided, 88.2%), item 2b (points used, 100%), item 2c (depths of insertion, 100%), item 2d (response sought, 76.5%), item 2e (needle stimulation, 100%), item 2f (needle retention time, 100%), item 2g (needle type, 94.1%), item 3a (number of treatment sessions, 100%), and item 3b (frequency and duration of treatment sessions, 100%). While, item 1c (the extent to which treatment was varied), item 2a (number of needle insertions per subject per session), and item 4a (details of other interventions) were not mentioned in the included studies. The STRICTA checklist is shown in Supplementary Table 5.

### 3.5. ALE meta-analysis results

#### 3.5.1. The immediate effect of acupuncture

**FIGURE 3:**
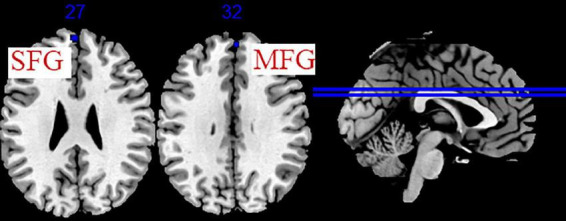
Regions of brain activation in patients with pre- to post- acupuncture (immediate effect). Blue represents brain deactivation regions.

Five studies evaluated the immediate effects of acupuncture on migraine (Peng, 2013; Xie, 2016; Ning et al., 2017; Fan, 2020; Liu S. et al., 2021). We extracted 82 foci with brain activation regions from 6 experiments, and 61 foci with brain deactivation regions from 5 experiments in patients with migraine after acupuncture treatment. No significant clusters with activation brain were detected between pre-acupuncture and post-acupuncture treatment. One significant cluster with deactivation brain was found between pre-acupuncture and post-acupuncture treatment: the left superior frontal gyrus (SFG, 168 mm^3^, BA9). The specific analysis results are presented in Table 4 and Figure 3.

**TABLE 4:** Changes of brain activation in patients with pre- to post- acupuncture.

Cluster number	Volume (mm^3^)	MNI coordinates			Peak ALE value	Area	Hemisphere	Brodmann area
		**X**	**Y**	**Z**				
**Post-acupuncture < Pre-acupuncture (Immediate effect)**								
1	168	−2	58	26	0.009318931	Frontal Lobe, SFG	L	9
		2	54	32	0.008554844	Frontal Lobe, MFG	L	6
**Post-acupuncture > Pre-acupuncture (Cumulative effect)**								
1	976	16	−20	6	0.014990999	THA	R	—
		16	−16	2	0.01456283	THA	R	—
		8	−26	2	0.008749179	THA, Pulvinar	R	—
2	400	24	42	42	0.014283381	Frontal Lobe, SFG	R	8
3	376	−36	−44	−36	0.012764244	Posterior Lobe, Cerebellar Tonsil	L	—
4	344	38	−12	20	0.011733903	INS	R	13
5	272	38	44	36	0.009829718	Frontal Lobe, MFG	R	8
6	224	36	−4	54	0.009671894	Frontal Lobe, PreCG	R	6
		34	−8	52	0.009619225	Frontal Lobe, PreCG	R	6
7	208	−2	34	6	0.009718803	Limbic Lobe, ACC	L	24
**Post-acupuncture < Pre-acupuncture (Cumulative effect)**								
1	744	54	−24	14	0.010470914	Temporal Lobe, TTG	R	41
		54	−16	14	0.009503565	Parietal Lobe, PoCG	R	43
		62	−30	12	0.009446988	Temporal Lobe, STG	R	42
		56	−30	10	0.009303252	Temporal Lobe, STG	R	41
2	328	−8	46	−16	0.013347376	Limbic Lobe, ACC	L	32
3	320	−28	−20	−16	0.010965006	Limbic Lobe, PHG	L	—
4	192	−46	−62	48	0.00994727	Parietal Lobe, IPL	L	40
		−44	−58	46	0.009831539	Parietal Lobe, IPL	L	40
5	160	−38	−74	−2	0.009893779	Occipital Lobe, IOG	L	19

SFG, superior frontal gyrus; MFG, medial frontal gyrus; THA, thalamus; INS: insula; PreCG, precentral gyrus; ACC, anterior cingulate; TTG: transverse temporal gyrus; PoCG, postcentral gyrus; STG, superior temporal gyrus; PHG, parahippocampal gyrus; IPL, inferior parietal lobule; IOG, inferior occipital gyrus; L, left; R, right.

#### 3.5.2. The cumulative effect of acupuncture

**FIGURE 4:**
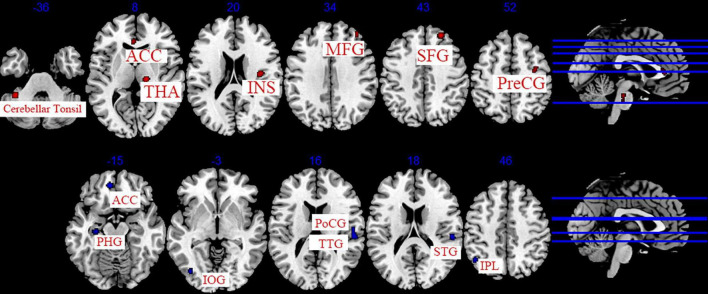
Regions of brain activation in patients with pre- to post- acupuncture (cumulative effect). Red represents brain activation regions; blue represents brain deactivation regions.

The data from 11 studies were pooled to investigate the cumulative effect of acupuncture on migraine (Zhao et al., 2014; Xie, 2016; Zhang, 2016; Cai, 2017; Li et al., 2017; Du, 2019; Wang, 2019; Zhang, 2019; Jia, 2021; Liu S. et al., 2021; Zhang Y. et al., 2021). We extracted the 77 foci with brain activation regions from 13 experiments and 83 foci with brain deactivation regions from 12 experiments in patients with migraine after acupuncture treatment. Seven activated clusters were found between pre-acupuncture and post-acupuncture treatment: (1) the right thalamus (THA, 976 mm^3^); (2) the right superior frontal gyrus (SFG, 400 mm^3^, BA8); (3) the left posterior lobe (376 mm^3^); (4) the right insula (INS, 344 mm^3^, BA13); (5) the right middle frontal gyrus (MFG, 272 mm^3^, BA8); (6) the right precentral gyrus (PreCG, 224 mm^3^, BA6); (7) the left anterior cingulate (ACC, 208 mm^3^, BA24). Five deactivated clusters were detected between pre-acupuncture and post-acupuncture treatment: (1) the right transverse temporal gyrus (TTG, 744 mm^3^, BA41); (2) the left anterior cingulate (ACC, 328 mm^3^, BA32); (3) the left parahippocampal gyrus (PHG, 192 mm^3^); (4) the left inferior parietal lobule (IPL, 192 mm^3^, BA40); (5) the left inferior occipital gyrus (IOG, 160 mm^3^, BA19). The specific analysis results are provided in Table 4 and Figure 4.

### 3.6. Sensitivity analysis

We conducted a sensitivity analysis on the immediate effect and cumulative effect of acupuncture, and the details are shown in Supplementary Table 6. Leave-one-out analysis of the immediate effect (post-acupuncture < pre-acupuncture) showed that the repeatability of the left SFG was up to 3/5 times of analysis, and MFG was up to 4/5 times of analysis. Leave-one-out analysis of the cumulative effect (post-acupuncture > pre-acupuncture) revealed that right THA, MFG was up to 12/13 times of analysis; right SFG, INS, PreCG, and left posterior lobe of the cerebellum, ACC was up to 11/13 times of analysis. Leave-one-out analysis of the cumulative effect (post-acupuncture < pre-acupuncture) showed that the repeatability of the right TTG, Postcentral gyrus (PoCG), Superior Temporal Gyrus (STG), and left ACC, PHG, IPL, IOG was up to 10/12 times of analysis.

## 4. Discussion

In the present study, we used the ALE method to perform a quantitative integration analysis of previously published original research and to identify the cerebral responses to acupuncture for migraine. The ALE analysis results of immediate acupuncture treatment showed that the deactivation regions located in the SFG and MFG. The ALE analysis results of cumulative acupuncture treatment revealed that the activation regions were the THA, SFG, posterior lobe of the cerebellum, INS, MFG, PreCG, ACC and the deactivation brain regions were the TTG, PoCG, STG, ACC, PHG, IPL, and IOG. The consistent results of immediate and cumulative change were the THA, INS, frontal lobe, parietal lobe, temporal lobe, occipital lobe, limbic system, and cerebellum. Pain is a multidimensional subjective experience generally including sensory (intensity, location), affective (unpleasantness, fear), and cognitive factors (memory, attention) (Schnitzler and Ploner, 2000). The brain processing network of pain can be divided into the medial and lateral pain systems, which are respectively involved in processing the affective-cognitive-evaluative aspects of pain, and the sensory-discriminative aspects (Friebel et al., 2011). Based on our findings, we found that several regions of the lateral and medial pain systems (including the THA, INS, ACC, SFG, MFG, and PoCG) participated in the cerebral responses to acupuncture for migraine.

The medial and ventrobasal parts of THA are separately involved in the medial and lateral pain pathways. In migraine pathogenesis, THA is considered as the relay center of ascending nociceptive information (Younis et al., 2019). Kim et al. (2021) observed that the regional fALFF values of the bilateral ventral posteromedial thalamus was positively correlated with the duration of migraine. Gu et al. (2018) discovered N-acetylaspartate/creatine increased in bilateral THA of migraineurs after acupuncture, which was significantly related with the headache intensity. The anterior part of the INS encodes the emotional processing of pain, and the posterior part encodes the intensity and lateralization of pain. Thus, the INS plays an integrative role in nociceptive processing. Zhang et al. (2022) found that the duration of migraine was negatively associated with gray matter (GM) alterations in the left INS. Cao et al. (2019) revealed that the INS was activated to integrate sensory and affective information and to produce analgesic effects during acupuncture stimulation. Our ALE meta-analysis showed that the activity of ACC (BA24) increased, while the activity of ACC (BA32) decreased after acupuncture in migraineurs. The ACC is considered to be involved in the affective-motivational component of pain (Price, 2000). Chen et al. (2021) discovered that the dynamic ALFF values in the ACC were negatively correlated with pain intensity in migraine. The ACC plays a different role in pain processing. Vogt et al. (1996) found that the increased regional cerebral blood flow (rCBF) in area 24’ of ACC may be involved in nocifensive reflex inhibition, and the reduced rCBF in area 32 of ACC may enhance pain perception in the surrounding cortex. Zhang et al. (2003) also discovered that the caudal part of the ACC (BA24) signal increased during the electrical acupoint stimulation, which positively related with the analgesic effect. Therefore, different parts of ACC may play different roles in the acupuncture-induced analgesic effect. The SFG is located at the superior part of the prefrontal cortex, which is responsible for emotion regulation (Cao et al., 2022). And the MFG is crucial to attention (Japee et al., 2015). The increased GM in the right SFG and the decreased GM in the left MFG were observed in migraineurs (Zhang et al., 2022). Zou et al. (2019) demonstrated that the decreased FC between the SFG and precuneus could restore to the healthy control level after acupuncture treatments. Russo et al. (2012) found that the FC between the MFG and the dorsal ACC reduced in migraineurs, and the decreased connectivity in the MFG was negatively related to the pain intensity of migraine attacks. The PoCG located in the primary somatosensory cortex (SI), which is involved in the adjustment of pain perception, including the positioning and recognition of pain intensity (Zhang S. et al., 2021). Wei et al. (2020) discovered that increased brain activity of the right PoCG is positively correlated with headache frequency in patients with migraine. Yang et al. (2012) also found that cerebral glucose metabolism decreased in the PoCG after acupuncture in migraine.

Besides, the TTG, STG, IPL, HPG, IOG, PreCG, and posterior lobe of the cerebellum are also involved in the analgesic effect of acupuncture. The temporal lobe is recognized as a region associated with multisensory integration. An analysis of voxel-based morphometric studies of migraine (Zhang et al., 2022) showed that GM increased in the bilateral temporal poles, the bilateral STG, the right SFG, and the left middle temporal gyrus (MTG) in migraine patients, and the frequency of migraine attacks was negatively associated with GM alteration in the left STG. This suggests that the temporal lobe is involved in pain regulation. Liu L. et al. (2022) revealed that the STG and MTG may be the key nodes linked to the multisensory processing of pain modulation in patients with migraine during acupuncture. It has been confirmed that the cerebellum plays an important role in pain processing and regulation (Moulton et al., 2010). Wang et al. (2016) observed that patients with migraine had significantly higher ALFF levels in the posterior lobe of the cerebellum in contrast with healthy controls. Zhang et al. (2022) revealed that the duration of migraine was negatively associated with GM alterations in the bilateral cerebellum (hemispheric lobule IX). Zhang et al. (2022) also found that GM increased in the PHG, and GM decreased in the IPL. Qin et al. (2020) discovered that reduced FC between the right IPL and right MFG, and the FC Z-scores between the ventral posterior nucleus (VPN) and right IPL were negatively related with pain intensity and disease duration in migraine. The PHG is involved in pain perception, pain modulation, and descending pain facilitation (Ruscheweyh et al., 2018). Yang et al. (2014) found that acupuncture could reduce brain glucose metabolism of PHG in migraine. Liu et al. (2012) observed that compared with healthy subjects, the PreCG exhibited abnormal centrality in both structural and FC networks in migraineurs, and negative correlations were observed between migraine duration and PreCG. Our results indicated that IOG also participated in acupuncture-induced analgesia, while few studies supported the finding.

There were 5 studies focused on the immediate effect of acupuncture and 11 studies on the cumulative effect. The results of ALE meta-analysis showed that both SFG and MFG were associated with immediate and cumulative effects, but the activity of SFG and MFG decreased after immediate acupuncture and increased after cumulative acupuncture. A possible explanation for the inconsistent results may be related to the number of included studies, which needs further investigation. Simultaneously, the neuroimaging results indicated that cumulative acupuncture treatment could induce a more extensive and remarkable cerebral response in contrast with single acupuncture treatment.

## 5. Strengths and limitations

In this meta-analysis, there are several strengths. First, instead of descriptive analysis, we used the ALE methodology to analyze the brain changes of acupuncture intervention in patients with migraine. Second, the present study analyzed the imaging studies on the immediate and cumulative effects of acupuncture in patients with migraine. Third, we comprehensively evaluated the methodological quality and the reporting quality of interventions with a modified version of checklist and STRICTA. Nevertheless, there are several limitations. First, due to the limitations of the analytical method, funnel plot was unavailable to detect the publication bias of the included studies. Second, we failed to compare the brain changes of patients with migraine after acupuncture with those in healthy individuals due to the incomplete information among included studies. Third, the STRICTA results showed that the protocol of acupuncture was not reported adequately, researchers should report in accordance with STRICTA to improve the reporting quality.

## 6. Conclusion

Acupuncture could activate multiple brain areas related with the regulation of pain conduction, processing, emotion, cognition, and other brain regions in patients with migraine. In the future, the combination of multiple imaging technologies might be a new approach to deeply investigate the central mechanism of acupuncture for migraine.

## Author contributions

JZ designed the protocol, conducted this review, and drafted the manuscript. JL pointed out the research question, designed the protocol, and drafted the manuscript. X-YG and X-BL screened the articles, collected and assessed the data. YZ analyzed the data. Y-XL, D-LZ, YF, ZZ, and R-JJ revised this manuscript, and provided constructive suggestions for this review. Y-XL provided guidance on data analysis. L-XG and H-RL provided clinical knowledge support for this review. JL pointed out the research question, and guided the whole process of this review. All authors reviewed and approved this review.

## Footnotes

1
https://inplasy.com/inplasy-2022-11-0026/

2
http://brainmap.org/ale/

3
https://www.nitrc.org/projects/mricron
