# Supplementary material for: The effects of acupuncture therapy in migraine: An activation likelihood estimation meta-analysis
Source: Front Neurosci. 2023 Jan 27;16:1097450. doi: 10.3389/fnins.2022.1097450 (PMC9911686; doi:10.3389/fnins.2022.1097450)
Supplement: Supplementary file 1 [file Table_1.DOCX]

**Supplementary Table 1 The full search strategies of all databases**

|  | **Coverage** | **Searches** | **Hits** |
| --- | --- | --- | --- |
| PubMed Database | Date of inception -August 18, 2022 | #1:(((((acupuncture[MeSH Terms]) OR (acupuncture Therapy[MeSH Terms])) OR (acupoint[MeSH Terms])) OR (acupuncture Point[MeSH Terms])) OR (electroacupuncture[MeSH Terms])) OR (electro-acupuncture[MeSH Terms])  #2:((((((acupuncture[Title/Abstract]) AND (electro-acupuncture[Title/Abstract])) AND (body needling[Title/Abstract])) OR (acupuncture Therapy[Title/Abstract])) OR (acupoint[Title/Abstract])) OR (acupuncture Point[Title/Abstract])) OR (electroacupuncture[Title/Abstract])  #3:#1 AND #2  #4:((((((((Functional magnetic resonance imaging[MeSH Terms]) OR (Functional MRI[MeSH Terms])) OR (fMRI[MeSH Terms])) OR (ALFF[Title/Abstract])) OR (fALFF[Title/Abstract])) OR (ReHo[Title/Abstract])) OR (amplitude of low-frequency fluctuation[Title/Abstract])) OR (fractional amplitude of low-frequency fluctuation[Title/Abstract])) OR (regional homogeneity[Title/Abstract])  #5:((migraine[MeSH Terms]) OR (Headache Disorders[MeSH Terms])) OR (Headache[MeSH Terms])  #6:(((headache[Title/Abstract]) OR (migraine[Title/Abstract])) OR (cephalgia[Title/Abstract])) OR (cephalalgia[Title/Abstract])  #7:#5 AND #6  #8:#3 AND #5 AND #7 | 21 |
| EMBASE Database | Date of inception -August 18, 2022 | #1:'functional magnetic resonance imaging'/exp OR 'fractional amplitude of low-frequency fluctuation'/exp OR 'amplitude of low-frequency fluctuation'/exp OR 'regional homogeneity'/exp  #2:alff:ti,ab,kw OR reho:ti,ab,kw OR falff:ti,ab,kw OR fmri:ti,ab,kw  #3:#1 AND #2  #4:'acupuncture'/exp OR 'electroacupuncture'/exp OR 'acupuncture point'/exp  #5:'headache'/exp OR 'migraine'/exp  #6:#3 ADN #4 AND #5 | 68 |
| Web of Science | Date of inception -August 18, 2022 | #1: TS=(ALFF OR fALFF OR ReHo OR amplitude of low-frequency fluctuation OR fractional amplitude of low-frequency fluctuation OR regional homogeneity OR Functional magnetic resonance imaging OR fMRI)  #2: TS=( Headache OR migraine OR cephalgia OR cephalalgia)  #3: TS=( acupuncture OR acupuncture Therapy OR electro-acupuncture OR electroacupuncture OR body needling OR acupuncture Point OR acupoint OR body needling)  #4: #3 AND #2 AND #1 | 54 |
| Cochrane Library | Date of inception - August 18,2022 | #1:MeSH descriptor: [magnetic resonance imaging] explode all trees  #2:(amplitude of low-frequency fluctuation):ti,ab,kw OR (fractional amplitude of low-frequency fluctuation):ti,ab,kw OR (regional homogeneity):ti,ab,kw  #3:(ALFF):ti,ab,kw OR (fALFF):ti,ab,kw OR (ReHo):ti,ab,kw OR (fMRI):ti,ab,kw  #4:#1 OR #2  #5:MeSH descriptor: [Acupuncture] explode all trees  #6:MeSH descriptor: [Electroacupuncture] explode all trees  #7:MeSH descriptor: [Acupuncture Points] explode all trees  #8:(body needling):ti,ab,kw OR (acupoint):ti,ab,kw OR (electro-acupuncture):ti,ab,kw OR (acupuncture Therapy):ti,ab,kw  #9:#5 OR #6 OR #7 OR #8  #10:MeSH descriptor: [Headache] explode all trees  #11:MeSH descriptor: [Migraine Disorders] explode all trees  #12:(cephalgia):ti,ab,kw OR (cephalalgia):ti,ab,kw  #13:#10 OR #11 OR #12  #14:#13 AND #4 AND #9 | 7 |
| China National Knowledge Infrastructure (CNKI, Chinese Database) | Date of inception - August 18, 2022 | SU=('针刺' + '针灸' + '电针') and SU=('头痛' + '偏头痛' ) and SU=('磁共振成像' + 'fMRI' + '低频振荡振幅' + 'ALFF' + '低频振荡振幅比率' + 'fALFF' + '局部一致性' + 'ReHo' ) | 68 |
| Chinese Biomedical Literature Database  (CBM, Chinese Database) | Date of inception - August 18, 2022 | #1:("偏头痛"[不加权:扩展]) OR "头痛"[不加权:扩展]  #2:(("针刺疗法"[不加权:扩展]) OR "针灸疗法"[不加权:扩展]) OR "电针"[不加权:扩展]  #3:"磁共振成像"[不加权:扩展]  #4:"ALFF"[全部字段:智能] OR "fALFF"[全部字段:智能] OR "ReHo"[全部字段:智能] OR "低频振荡振幅"[全部字段:智能] OR "低频振荡振幅比率"[全部字段:智能] OR "局部一致性"[全部字段:智能] OR "fMRI"[全部字段:智能]  #5: (#4) OR (#3)  #6: (#5) AND (#2) AND (#1) | 22 |
| China Science and Technology Journal Database (VIP, Chinese Database) | Date of inception - August 18, 2022 | R=(ALFF OR fALFF OR ReHo OR 低频振荡振幅 OR 低频振荡振幅比率 OR 局部一致性 OR fMRI OR 磁共振成像) AND M=(针灸 OR 针刺 OR 电针) AND M=(头痛 OR 偏头痛) | 20 |
| Wanfang Database  (WF, Chinese Database) | Date of inception -August 18,2022 | 主题:(ALFF OR fALFF OR ReHo OR 低频振荡振幅 OR 低频振荡振幅比率 OR 局部一致性 OR MRI OR 磁共振成像) and 主题:(针灸 OR 针刺 OR 电针) and 主题:(疼痛 OR 痛 OR 炎 ) | 78 |

**Supplementary Table 2. Reasons for exclusion of full manuscripts screened and not included in systematic review**

| **Manuscript** | **Reasons** |
| --- | --- |
| 基于fMRI探讨针刺治疗偏头痛疗效差异的中枢机制 | 1 |
| 针刺治疗偏头痛的临床疗效评价及相关静息态功能磁共振成像研究 | 1 |
| Cerebral fractional amplitude of low-frequency fluctuations may predict headache intensity improvement following acupuncture treatment in migraine patients | 2 |
| 针刺少阳经穴对偏头痛患者疼痛矩阵相关脑区局部一致性的影响 | 2 |
| 基于静息态fMRI观察针刺不同组穴对偏头痛患者大脑局部一致性影响的研究 | 2 |
| 针刺少阳经特定穴对偏头痛患者脑功能动态影响的研究 | 2 |
| 针刺足临泣对无先兆偏头痛患者皮层局部一致性影响的研究 | 2 |
| 基于"根结"理论辨经取穴针刺治疗无先兆偏头痛患者的临床疗效及其调控脑区研究 | 2 |
| 针刺少阳经非特定穴治疗偏头痛的临床效应及中枢机制研究 | 2 |
| 基于磁共振静息态的针刺足临泣对偏头痛疼痛相关脑网络影响的研究 | 2 |
| 针刺少阳经腧穴治疗偏头痛即时效应和累积效应的大脑局部一致性研究 | 2 |
| 针刺足临泣对偏头痛低频振荡振幅的影响 | 2 |
| 不同远近配穴法治疗月经性偏头痛的脑功能局部一致性分析 | 2 |
| 针刺少阳经腧穴治疗偏头痛即时效应和累积效应的大脑局部一致性研究 | 2 |
| 基于大脑疼痛感知网络研究针刺治疗月经性无先兆偏头痛患者的中枢镇痛机制 | 2 |
| 针刺调节月经性偏头痛患者负性情绪的大脑局部功能研究 | 2 |
| Regulation of cerebral regional homogeneity exerted by puncturing hao-yang acupoints for migraine patients | 3 |
| Analysis on regional homogeneity of resting brain during balance acupuncture-induced analgesic effect in migraine patients without aura | 3 |
| 基于fMRI技术针刺太冲穴对不同状态受试者脑功能的影响研究 | 3 |
| 针刺治疗偏头痛疗效差异的中枢机制及单核苷酸多态性研究 | 3 |
| Reasons for exclusion classified as: 1. Different analyse methods; 2. Reduplicate articles; 3. No data available. | |

**Supplementary Table 3 Results for objective assessment of methodological quality of individual studies**

| **Study** | **Category 1: Sample characteristics** | | | | | **Category 2: Methodology and reporting** | | | | | | | | **total** |
| --- | --- | --- | --- | --- | --- | --- | --- | --- | --- | --- | --- | --- | --- | --- |
|  | 1 | 2 | 3 | 4 | 5 | 1 | 2 | 3 | 4 | 5 | 6 | 7 | 8 |  |
| Peng, 2013 | 1 | 2 | 1 | 0 | 2 | 3 | 1 | 1 | 1 | 1 | 1 | 1 | 1 | 16 |
| Zhao, 2014 | 1 | 2 | 0 | 3 | 2 | 3 | 1 | 1 | 1 | 1 | 1 | 1 | 1 | 18 |
| Xie,  2016 | 1 | 2 | 0 | 2 | 2 | 3 | 1 | 1 | 1 | 1 | 1 | 1 | 1 | 17 |
| Zhang,  2016 | 1 | 2 | 1 | 3 | 2 | 3 | 1 | 1 | 1 | 1 | 1 | 1 | 1 | 19 |
| Cai,  2017 | 1 | 2 | 0 | 3 | 2 | 3 | 1 | 1 | 1 | 1 | 1 | 1 | 1 | 18 |
| Li,  2017 | 1 | 2 | 1 | 3 | 2 | 3 | 1 | 1 | 1 | 1 | 1 | 1 | 1 | 19 |
| Ning, 2017 | 1 | 2 | 1 | 3 | 2 | 3 | 1 | 1 | 1 | 1 | 1 | 1 | 1 | 19 |
| Du,  2019 | 1 | 2 | 0 | 3 | 1 | 3 | 1 | 1 | 1 | 1 | 1 | 1 | 1 | 17 |
| Wang,  2019 | 1 | 2 | 1 | 3 | 0 | 3 | 1 | 1 | 1 | 1 | 1 | 1 | 1 | 17 |
| Zhang, 2019 | 1 | 2 | 0 | 2 | 2 | 3 | 1 | 1 | 1 | 1 | 1 | 1 | 1 | 17 |
| Fan,  2020 | 1 | 2 | 0 | 3 | 0 | 3 | 1 | 1 | 1 | 1 | 1 | 1 | 1 | 16 |
| Jia,  2021 | 1 | 2 | 0 | 4 | 2 | 3 | 1 | 1 | 1 | 1 | 1 | 1 | 1 | 19 |
| Liu,  2021 | 1 | 2 | 1 | 3 | 2 | 3 | 1 | 1 | 1 | 1 | 1 | 1 | 1 | 19 |
| Zhang, 2021 | 1 | 2 | 0 | 2 | 2 | 3 | 1 | 1 | 1 | 1 | 1 | 1 | 1 | 17 |

**Supplementary Table 4 Criteria for objective assessment of methodological quality of individual studies**

| **Category 1: Sample characteristics (10)** |
| --- |
| 1. Patients were evaluated with specific standardised diagnostic criteria (1) |
| 1. Important demographic data (age and gender) were reported with mean (or median) and standard deviations (or range)) (2) |
| 1. Healthy comparison subjects were evaluated to exclude psychiatric and medical illnesses and demographic data was reported (1) |
| 1. Important clinical variables (e.g. illness duration, onset time, medication status, pain scores) were reported with mean (or median) and standard deviations (or range)) (4) |
| 1. Sample size per group > 10 (2) |
| **Category 2: Methodology and reporting (10)** |
| 1. Whole brain analysis was automated with no a-priori regional selection (3) |
| 1. Magnet strength at least 1.5T (1) |
| 1. At least 5 minutes of resting state acquisition (1) |
| 1. Whole brain coverage of resting scans (1) |
| 1. The acquisition and preprocessing techniques were clearly described so that they could be reproduced (1) |
| 1. Coordinates reported in a standard space (1) |
| 1. Significant results are reported after correction for multiple testing using a standard statistical procedure (FDR, FWE or permutation-based methods) (1) |
| 1. Conclusions were consistent with the results obtained and the limitations were discussed (1) |

**Supplementary Table 5 Results for STRICTA checklist**

| **Studies** | **1. Acunpuncture rationale** | | | **2. Details of needling** | | | | | | | **3. Treatment regimen** | | **4.**  **Cointerventions** | | **5.**  **Practitioner background** | **6. Control or comparator interventions** | |
| --- | --- | --- | --- | --- | --- | --- | --- | --- | --- | --- | --- | --- | --- | --- | --- | --- | --- |
|  | **1a** | **1b** | **1c** | **2a** | **2b** | **2c** | **2d** | **2e** | **2f** | **2g** | **3a** | **3b** | **4a** | **4b** | **5** | **6a** | **6b** |
| Peng,  2013 | Yes | Yes | No | No | Yes | Yes | Yes | Yes | Yes | Yes | Yes | Yes | No | Yes | No | No | No |
| Zhao,  2014 | Yes | Yes | No | No | Yes | Yes | Yes | Yes | Yes | Yes | Yes | Yes | No | No | Yes | Yes | Yes |
| Xie,  2016 | Yes | Yes | No | No | Yes | Yes | Yes | Yes | Yes | Yes | Yes | Yes | No | Yes | No | No | Yes |
| Zhang,  2016 | Yes | Yes | No | No | Yes | Yes | Yes | Yes | Yes | Yes | Yes | Yes | No | No | Yes | No | No |
| Ning,  2017 | Yes | Yes | No | No | Yes | Yes | Yes | Yes | Yes | Yes | Yes | Yes | No | No | Yes | No | No |
| Li,  2017 | Yes | Yes | No | No | Yes | Yes | Yes | Yes | Yes | No | Yes | Yes | No | No | Yes | Yes | Yes |
| Cai,  2017 | Yes | No | No | No | Yes | Yes | Yes | Yes | Yes | Yes | Yes | Yes | No | No | Yes | No | Yes |
| Du,  2019a | Yes | Yes | No | No | Yes | Yes | No | Yes | Yes | Yes | Yes | Yes | No | No | No | No | No |
| Du,  2019b | Yes | Yes | No | No | Yes | Yes | No | Yes | Yes | Yes | Yes | Yes | No | No | No | No | No |
| Wang,  2019a | Yes | Yes | No | No | Yes | Yes | Yes | Yes | Yes | Yes | Yes | Yes | No | No | Yes | Yes | Yes |
| Wang,  2019b | Yes | Yes | No | No | Yes | Yes | Yes | Yes | Yes | Yes | Yes | Yes | No | No | Yes | Yes | Yes |
| Zhang,  2019 | Yes | Yes | No | No | Yes | Yes | Yes | Yes | Yes | Yes | Yes | Yes | No | No | No | Yes | Yes |
| Fan,  2020a | Yes | Yes | No | No | Yes | Yes | No | Yes | Yes | Yes | Yes | Yes | No | No | No | Yes | Yes |
| Fan,  2020b | Yes | Yes | No | No | Yes | Yes | No | Yes | Yes | Yes | Yes | Yes | No | No | No | Yes | Yes |
| Jia,  2021 | Yes | Yes | No | No | Yes | Yes | Yes | Yes | Yes | Yes | Yes | Yes | No | No | No | Yes | Yes |
| Liu,  2021 | Yes | No | No | No | Yes | Yes | Yes | Yes | Yes | Yes | Yes | Yes | No | No | Yes | No | No |
| Zhang,  2021 | Yes | Yes | No | No | Yes | Yes | Yes | Yes | Yes | Yes | Yes | Yes | No | No | Yes | Yes | Yes |
| Note: 1a, style of acupuncture; 1b, reasoning for treatment provided, based on historical context, literature sources, and/or consensus methods, with references where appropriate; 1c, extent to which treatment was varied; 2a, number of needle insertions per subject per session (mean and range where relevant); 2b,points used (unilateral/bilateral); 2c, depths of insertion (eg, cun or tissue level); 2d, responses elicited (eg, de qi or twitch response); 2e, needle stimulation (eg, manual or electrical); 2f, needle retention time; 2g, needle type (diameter, length, and manufacturer or material); 3a, number of treatment sessions; 3b, frequency of treatment; 4a, details of other interventions (eg, moxibustion, cupping, herbs, exercises, lifestyle advice); 4b, setting and context of treatment, including instructions to practitioners, and information and explanations to patients; 5, Description of participating acupuncturists (qualification or professional affiliation, years in acupuncture practice, other relevant experience); 6a, Rationale for the control or comparator in the context of the research question, with sources that justify this choice; 6b, Precise description of the control or comparator. If sham acupuncture or any other type of acupuncture-like control is used, provide details as for Items 1 to 3 above; No, no details report; Yes, details reported. | | | | | | | | | | | | | | | | | |

**Supplementary Table 6 Sensitive analyses of pre-post acupuncture contrast ALE meta-analyses**

| **Discarded Article** | **Post-acupuncture < Pre-acupuncture (Immediate Effect)** | | | | | | | |
| --- | --- | --- | --- | --- | --- | --- | --- | --- |
|  | SFG_L | | | | MFG_L | | | |
| Peng ,2013 | N | | | | Y | | | |
| Xie ,2016 | N | | | | N | | | |
| Ning,2017 | Y | | | | Y | | | |
| Fan,2020a | Y | | | | Y | | | |
| Fan,2020b | Y | | | | Y | | | |
|  | 3 | | | | 4 | | | |
| **Discarded Article** | **Post-acupuncture > Pre-acupuncture (Cumulative Effect)** | | | | | | | |
|  | **THA_R** | **SFG_R** | **Posterior Lobe_L** | **INS_R** | | **MFG_R** | **PreCG_R** | **ACC_L** |
| Zhao,2014 | N | Y | Y | N | | Y | N | N |
| Xie,2016 | Y | Y | N | N | | Y | N | N |
| Zhang,2016 | Y | Y | Y | Y | | Y | Y | Y |
| Cai,2017 | Y | Y | N | Y | | Y | Y | Y |
| Li,2017 | Y | Y | Y | Y | | Y | Y | Y |
| DU,2017a | Y | Y | Y | Y | | Y | Y | Y |
| DU,2017b | Y | Y | Y | Y | | Y | Y | Y |
| Wang,2019a | Y | N | Y | Y | | Y | Y | Y |
| Wang,2019b | Y | Y | Y | Y | | Y | Y | Y |
| Zhang,2019 | Y | Y | Y | Y | | Y | Y | Y |
| Jia,2021b | Y | Y | Y | Y | | Y | Y | Y |
| Liu,2021 | Y | Y | Y | Y | | Y | Y | Y |
| Zhang,2021 | Y | N | Y | Y | | N | Y | Y |
|  | 12 | 11 | 11 | 11 | | 12 | 11 | 11 |
| **Discarded Article** | **Post-acupuncture < Pre-acupuncture (Cumulative Effect)** | | | | | | | |
|  | **TTG_R** | **PoCG_R** | **STG_R** | **ACC_L** | | **PHG_L** | **IPL_L** | **IOG_L** |
| Zhao,2014 | Y | Y | Y | Y | | N | N | Y |
| Xie,2016 | N | Y | Y | Y | | N | Y | Y |
| Zhang,2016 | Y | Y | Y | Y | | Y | N | N |
| Cai,2017 | Y | Y | Y | Y | | Y | Y | Y |
| Li,2017 | Y | Y | Y | Y | | Y | Y | Y |
| DU,2019a | Y | Y | Y | Y | | Y | Y | Y |
| DU,2019b | Y | N | N | N | | Y | Y | Y |
| Wang,2019a | Y | Y | Y | Y | | Y | Y | N |
| Wang ,2019b | Y | Y | Y | N | | Y | Y | Y |
| Jia,2021a | Y | Y | Y | Y | | Y | Y | Y |
| Jia,2021b | Y | Y | Y | Y | | Y | Y | Y |
| Zhang,2021 | N | N | N | Y | | Y | Y | Y |
|  | 10 | 10 | 10 | 10 | | 10 | 10 | 10 |
| R, Right; L, Left; Y, Yes; N, No; SFG, Superior Frontal Gyrus; MFG, Medial Frontal Gyrus; PreCG, Precentral Gyrus; ACC, Anterior Cingulate; TTG, Transverse Temporal Gyrus; PoCG, Postcentral Gyrus; STG, Superior Temporal Gyrus; PHG, Parahippocampal Gyrus; IPL, Inferior Parietal Lobule; IOG, Inferior Occipital Gyrus; THA, Thalamus; INS, Insula | | | | | | | | |
